# Benchmarking paediatric living donor liver transplantation for establishing standards to expand access and save lives: retrospective global cohort study

**DOI:** 10.1093/bjsopen/zrag154

**Published:** 2026-09-25

**Authors:** Zhihao Li, Omid Madadi-Sanjani, Yuan Liu, Dimitri A Raptis, Vasanthakumar Gunasekaran, Ashwin Rammohan, Seisuke Sakamoto, Hajime Uchida, Piotr Kaliciński, Marek Stefanowicz, Nicolas Goldaracena, Cornelius J van Beekum, Felix J Krendl, Itsuko Chih-Yi Chen, Bryanna N Domenick, Alfred Kow, Kaihang Wang, Yuji Soejima, Katryn N Furuya, George V Mazariegos, Vidyadhar Padmakar Mali, Stephen C Dunn, Kim M Olthoff, Elizabeth B Rand, Ian Leipnitz, Chao-Long Chen, Deniz Balci, Elvan Onur Kirimker, Silvio Nadalin, Stefan Schneeberger, Debra L Sudan, Moritz Schmelzle, Uta Herden, Lutz Fischer, Blayne A Sayed, Vicky L Ng, Grzegorz Kowalewski, Mureo Kasahara, Mohamed Rela, Dieter C Broering, Qiang Xia, Pierre-Alain Clavien

**Affiliations:** Department of Surgery and Transplantation, University of Zurich, Zurich, Switzerland; Department of Visceral Transplantation, University Medical Centre Hamburg-Eppendorf, Hamburg, Germany; Department of Liver Surgery, Renji Hospital Affiliated to Shanghai Jiao Tong University School of Medicine, Shanghai, China; Organ Transplant Centre of Excellence, King Faisal Specialist Hospital and Research Centre, Riyadh, Saudi Arabia; Institue of Liver Disease and Transplantation, Dr Rela Institute and Medical Centre, Chennai, India; Institue of Liver Disease and Transplantation, Dr Rela Institute and Medical Centre, Chennai, India; Organ Transplantation Centre, National Centre for Child Health and Development (NCCHD), Tokyo, Japan; Organ Transplantation Centre, National Centre for Child Health and Development (NCCHD), Tokyo, Japan; Department of Paediatric Surgery and Organ Transplantation, The Children's Memorial Health Institute, Warsaw, Poland; Department of Paediatric Surgery and Organ Transplantation, The Children's Memorial Health Institute, Warsaw, Poland; Department of Surgery, Division of Abdominal Transplant Surgery, University Health Network, Toronto, Ontario, Canada; Department of General, Visceral and Transplant Surgery, Hannover Medical School, Hannover, Germany; Department of Visceral, Transplant and Thoracic Surgery, Centre for Operative Medicine, Medical University of Innsbruck, Innsbruck, Austria; Department of Surgery, Kaohsiung Chang Gung Memorial Hospital, Kaohsiung, Taiwan; Division of Gastroenterology, Hepatology and Nutrition, Department of Pediatrics, Children's Hospital of Philadelphia, Philadelphia, Pennsylvania, USA; Department of Paediatric Surgery, National University Hospital, Singapore; University of Zurich, Zurich, Switzerland; Division of Gastroenterological, Hepato-Biliary-Pancreatic, Transplantation and Paediatric Surgery, Department of Surgery, Shinshu University School of Medicine, Matsumoto, Japan; Department of Paediatrics, School of Medicine and Public Health, University of Wisconsin–Madison, Madison, Wisconsin, USA; Hillman Centre for Paediatric Transplantation, UPMC Children's Hospital of Pittsburgh, Pittsburgh, Pennsylvania, USA; Department of Paediatric Surgery, National University Hospital, Singapore; Department of Solid Organ Transplantation, Alfred I. DuPont Hospital for Children, Wilmington, Delaware, USA; Division of Gastroenterology, Hepatology and Nutrition, Department of Pediatrics, Children's Hospital of Philadelphia, Philadelphia, Pennsylvania, USA; Department of Surgery, Penn Transplant Institute, Perelman School of Medicine, University of Pennsylvania, Philadelphia, Pennsylvania, USA; Division of Gastroenterology, Hepatology and Nutrition, Department of Pediatrics, Children's Hospital of Philadelphia, Philadelphia, Pennsylvania, USA; Liver Transplant Unit, University of Auckland, Auckland, New Zealand; Department of Surgery, Kaohsiung Chang Gung Memorial Hospital, Kaohsiung, Taiwan; Department of General Surgery, Bahçeşehir University Faculty of Medicine, İstanbul, Turkey; Department of General Surgery, Ankara University Faculty of Medicine, Ankara, Turkey; Department of General Visceral and Transplant Surgery, Eberhard Karls University of Tübingen, Tübingen, Germany; Department of Visceral, Transplant and Thoracic Surgery, Centre for Operative Medicine, Medical University of Innsbruck, Innsbruck, Austria; Division of Abdominal Transplantation, Department of Surgery, Duke University Medical Centre, Durham, North Carolina, USA; Department of General, Visceral and Transplant Surgery, Hannover Medical School, Hannover, Germany; Department of Visceral Transplantation, University Medical Centre Hamburg-Eppendorf, Hamburg, Germany; Department of Visceral Transplantation, University Medical Centre Hamburg-Eppendorf, Hamburg, Germany; Department of Surgery, Division of Abdominal Transplant Surgery, University Health Network, Toronto, Ontario, Canada; Division of Gastroenterology, Hepatology and Nutrition, Transplant and Regenerative Medicine Centre, The Hospital for Sick Children, Toronto, Ontario, Canada; Division of Gastroenterology, Hepatology and Nutrition, Transplant and Regenerative Medicine Centre, The Hospital for Sick Children, Toronto, Ontario, Canada; Department of Paediatric Surgery and Organ Transplantation, The Children's Memorial Health Institute, Warsaw, Poland; Organ Transplantation Centre, National Centre for Child Health and Development (NCCHD), Tokyo, Japan; Institue of Liver Disease and Transplantation, Dr Rela Institute and Medical Centre, Chennai, India; Organ Transplant Centre of Excellence, King Faisal Specialist Hospital and Research Centre, Riyadh, Saudi Arabia; Department of Liver Surgery, Renji Hospital Affiliated to Shanghai Jiao Tong University School of Medicine, Shanghai, China; Department of Surgery and Transplantation, University of Zurich, Zurich, Switzerland; Wyss Zurich Translational Centre, ETH Zurich and University of Zurich, Zurich, Switzerland

**Keywords:** paediatric liver transplantation, technical variant grafts

## Abstract

**Background:**

Paediatric liver transplantation increasingly incorporates technical variant grafts including living donor liver transplantation (LDLT) to address donor shortages. However, the technical complexity of LDLT and regional outcome variations remain challenging. In settings with such variation and ongoing debate, standardized benchmarks may guide decision-making for broader implementation. This study establishes benchmark cut-off values for paediatric LDLT outcomes to provide a validated framework for quality assessment and safe programme expansion.

**Methods:**

An international analysis was conducted of paediatric patients who underwent LDLT at 19 centres between 2019 and 2023. Benchmark cut-off values were derived from ‘ideal’ patients at seven high-volume institutions, representing the best achievable results under optimal conditions. Surgical complications were graded using the Clavien–Madadi classification, compared against the standard Clavien–Dindo system for accuracy.

**Results:**

In all, 2665 patients were included, of which 1988 counted as ideal patients. The median recipient age was 10 (interquartile range (i.q.r.) 6–26) months with median weight of 8.0 (i.q.r. 6.6–11.9) kg. Biliary atresia was the most common diagnosis (68.8%), and most recipients (90.9%) received a left lateral segment graft. The 75th percentile of median centre values were used to define the following benchmark cut-off values: hepatic artery thrombosis ≤ 5%, bile leak ≤ 12%, biliary anastomotic stricture ≤ 6%, and 1-year graft loss or mortality ≤ 6%. Higher centre volume was associated with more selective patient inclusion and fewer major (particularly biliary) complications. An inverse correlation between case volume and failure-to-rescue rate highlighted the critical role of multidisciplinary perioperative management. The Clavien–Madadi classification proved superior to the Clavien–Dindo system, which overclassified grade III complications.

**Conclusions:**

This study provides a framework to guide safe programme expansion, improve surgical quality, and reduce waitlist mortality.

## Teaser

This international study of 2665 children undergoing living donor liver transplantation (LDLT) at 19 centres across five continents established the first benchmark cut-off values for paediatric LDLT, derived from 1988 ideal patients at seven high-volume institutions. Low-volume centres failed to meet these thresholds for major biliary and vascular complications and had correspondingly poorer graft survival, whereas the novel Clavien–Madadi classification more accurately reflected complication severity in children than the Clavien–Dindo system. These benchmarks provide reference standards to guide quality assessment and support the safe, broader expansion of paediatric LDLT programmes.

## Introduction

Paediatric liver transplantation is a rapidly advancing field worldwide. However, securing high-quality, size-appropriate allografts remains a challenge and a key contributor to waitlist mortality^1^. To address this, the donor pool has expanded with technical variant grafts (TVGs), including living donor and split/reduced deceased donor grafts. Among these, living donor liver transplantation (LDLT) offers an alternative to deceased donation and has been associated with superior outcomes^2,3^. Nevertheless, LDLT poses technical challenges and entails donor risk, limiting its widespread adoption. LDLT practice varies worldwide: rates in Europe have remained stable at 30–35%^4^, whereas in Asia LDLT is the standard^5^. In the USA, paediatric LDLT increased from 9.9% in 2012 to a peak of 16.5% in 2022^6^, with little change since.

As the North American paediatric transplant community seeks to reduce waitlist mortality, an ongoing debate questions whether all candidates should have access to LDLT (and whether all centres should be required to offer it) or whether improved deceased donor graft utilization should be prioritized^7,8^. High-volume centres with extensive TVG experience have demonstrated significantly reduced paediatric waitlist mortality^9,10^. However, regional variations in TVG use and the expertise required underscore the need for standardized quality assessment in paediatric liver transplantation. Benchmarking is a validated method for measuring surgical quality and comparing institutional performance. It defines benchmark cut-off values based on outcomes of low-risk patients treated at expert centres, representing the best achievable results under optimal conditions for a given procedure. Although benchmarks exist for adult LDLT^11^, none currently guide paediatric LDLT or other paediatric procedures. Establishing such standards is essential to inform the debate on expanding paediatric LDLT programmes, set quality targets for training, and support broader implementation. In this context, the Clavien–Madadi classification, an adaptation of the Clavien–Dindo system for paediatric surgery, grades complications by the invasiveness of the intervention rather than the need for anaesthesia, preventing overclassification of minor events^12^. However, the Clavien–Madadi classification system has not yet been validated in the paediatric liver transplant population.

To address these gaps, this international study establishes the first benchmarks for paediatric LDLT and applies and comparatively evaluates the Clavien–Madadi classification in this setting. The findings aim to guide quality improvement and support the evolution of paediatric LDLT worldwide.

## Methods

### Study design

This study included consecutive paediatric patients (age < 18 years) who underwent paediatric LDLT between 1 January 2019 and 31 December 2023. Patients who underwent retransplantation or multiorgan transplantation were excluded. Benchmarks were established using standardized methodology^13^. High-volume benchmark centres were defined as those which performed ≥ 20 paediatric LDLT procedures per year or ≥ 100 LDLT procedures over 5 years, had previous publications on paediatric liver transplantation, and maintained a prospective patient database with at least 1 year of follow-up. Low-volume centres (≤ 20 procedures per year) were included as the control group. The benchmark cohort included patients with body weight > 5 kg, excluding those with paediatric acute liver failure, liver tumours, portal vein thrombosis, dialysis dependency, congenital heart disease, or hepatopulmonary syndrome before transplantation. These criteria were selected based on known prognostic factors in paediatric liver recipients, because such high-risk clinical features could confound the technical assessment of surgical performance^14–17^. Patients who did not meet benchmark criteria formed the non-benchmark cohort. The primary endpoints were the benchmark values.

Ethical approval for the study was obtained from the Cantonal Ethics Commission of Zurich, Switzerland (BASEC 2024-01544) and each participating centre.

### Data collection

Data were collected using a standardized Microsoft^®^ Excel (Microsoft, Redmond, WA, USA) spreadsheet with clearly defined variables and outcome definitions, distributed to all participating centres before data entry. The file was password protected, and completed deidentified sheets were returned to the coordinating centre via encrypted transfer, ensuring uniform event classification and consistent reporting across centres.

### Definition of benchmark values and other variables

Fourteen outcome parameters were selected to establish benchmark values. Intraoperative benchmarks included operation duration and intraoperative blood transfusion. Postoperative benchmarks, evaluated within the first year after liver transplantation, included bowel perforation, intra-abdominal bleeding, any biliary complication, bile leak, biliary anastomotic stricture, hepatic artery thrombosis, portal vein thrombosis, unplanned readmission, major morbidity (Clavien–Dindo grade ≥ IIIa)^18^, graft loss, and mortality. Morbidity was also graded using the novel Clavien–Madadi classification^12^. Where the Clavien–Dindo system differentiates grade IIIa and IIIb complications by the need for general anaesthesia, the Clavien–Madadi classification instead bases grading on the invasiveness of the intervention. Thus, minor procedures performed under anaesthesia (for example, drain placement or wound revision) are classified as grade IIIa, whereas only major surgical interventions such as laparotomy or thoracotomy are considered grade IIIb. In addition, although traditional systems grade only the most severe complication, the Comprehensive Complication Index (CCI^®^) integrates all experienced complications. Calculated as the sum of all complications weighted by clinical severity, the CCI^®^ formula provides a continuous scale from 0 to 100 to capture cumulative morbidity in a single patient^19^. Bile leaks were defined according to the International Study Group for Liver Surgery criteria^20^. Biliary strictures were diagnosed based on the observation of anastomosis narrowing in conjunction with graft dysfunction. Failure to rescue was defined as graft loss or death within 1 year following a major surgical complication.

### Statistical analysis

Descriptive statistics are presented as the median with interquartile range (i.q.r.) for continuous variables and as counts with percentages for categorical variables. Continuous variables were compared using the Mann–Whitney *U* test, whereas categorical variables were analysed using χ^2^ tests. Benchmark cut-off values were defined as the 75th percentile of the median values across all benchmark centres for each negative outcome indicator. Pearson correlation coefficients (*R*^2^) were calculated to evaluate the association between centre characteristics and outcomes. To ensure that no single high-volume institution disproportionately influenced the benchmark thresholds, each benchmark cut-off value was derived from centre-level median values rather than pooled patient-level data, so that every benchmark centre contributed equally to the 75th-percentile calculation regardless of its case volume. For the same reason, all centre-level correlation analyses treated each centre as a single observation.

Statistical analyses were conducted using R 4.4.3 (R Foundation for Statistical Computing, Vienna, Austria). Two-tailed *P* < 0.05 was considered statistically significant for all analyses.

## Results

### Participating centres and study population

**Figure 1:**
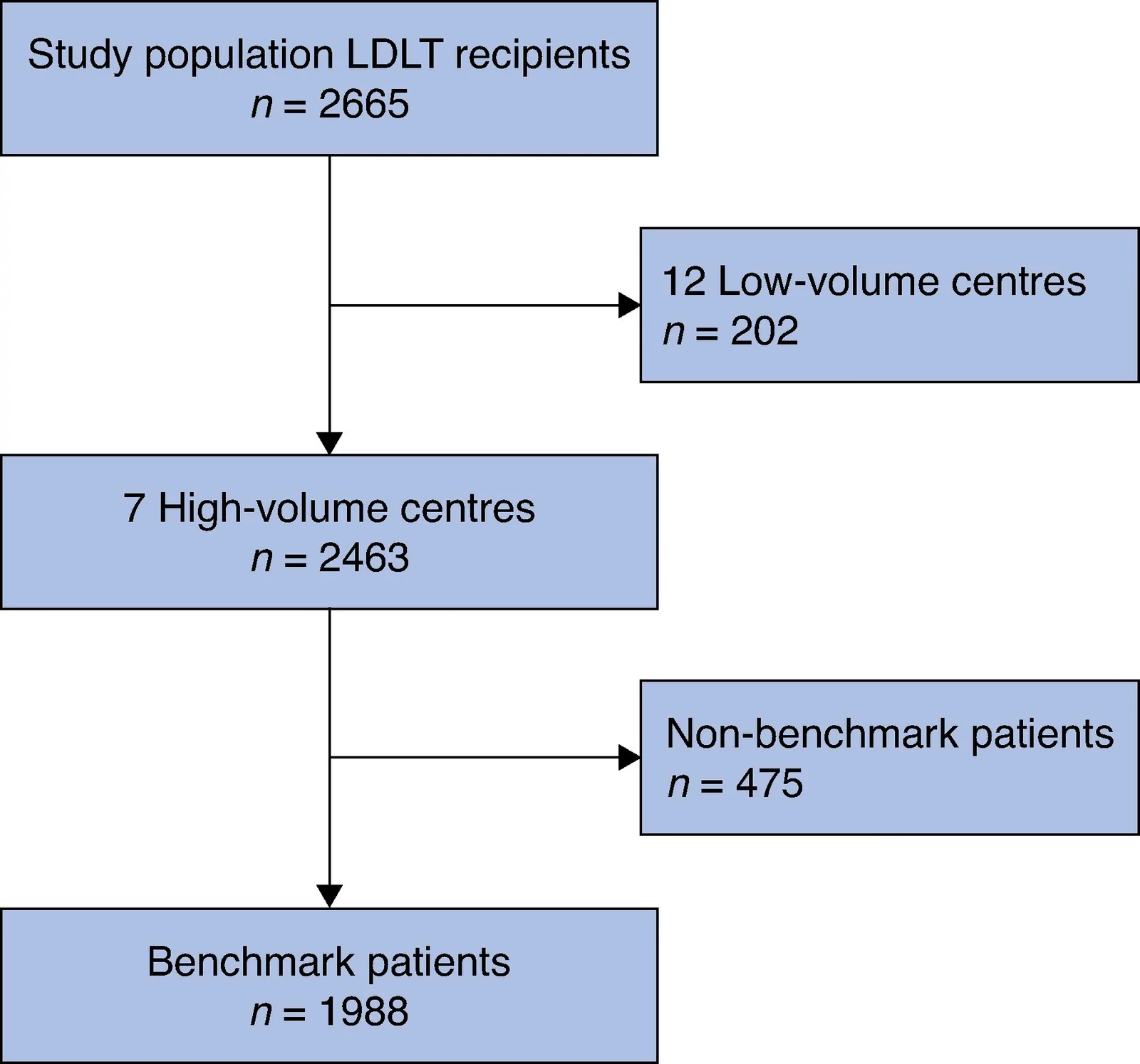
Study population structure and patient flow LDLT, living donor liver transplantation.

In all, 19 centres participated in the study, including seven high-volume centres. Centres were recruited through an international collaborative of paediatric liver transplant programmes maintaining prospective databases, with participation contingent on the ability to provide consecutive, prospectively collected data meeting the predefined quality criteria. The participating centres spanned five continents: Asia (China, Japan, India, Taiwan, Singapore), Europe (Switzerland, Germany, Poland, Austria, Turkey), North America (USA, Canada), the Middle East (Saudi Arabia), and Oceania (New Zealand). This study analysed paediatric LDLT exclusively; deceased donor recipients were not included, and annual caseload was defined by paediatric LDLT volume. The proportion of benchmark patients per centre ranged from 25% to 91% (*Fig. S1*). This wide variability in the proportion of benchmark patients reflects genuine differences in case mix and referral patterns across centres rather than data inconsistency. Across all centres, 2665 patients underwent LDLT, 2463 at high-volume centres (*Fig. 1*). The single largest centre contributed 45% of the overall cohort (*Fig. S1*). Baseline characteristics for LDLT recipients from low-volume centres are summarized in *Table S1*. Among the 2463 LDLT patients from high-volume centres, 1988 (80.7%) met the benchmark criteria. Their characteristics are summarized in *Table 1*. The median recipient age was 10 (i.q.r. 6–26) months with a median weight of 8.0 (i.q.r. 6.6–11.9) kg. Biliary atresia was the most common diagnosis (68.8%), followed by metabolic diseases (18.0%) and other cholestatic conditions (13.3%). The median paediatric end-stage liver disease score was 16 (i.q.r. 8–23). Donors had a median age of 32 (i.q.r. 29–36) years. Most recipients (90.9%) received a left lateral segment graft; 7.9% received a left lobe and 1.2% received a right lobe. The median graft-to-recipient weight ratio was 3.0 (i.q.r. 2.2–3.8). The median cold and warm ischaemia times were 29 (i.q.r. 20–71) and 37 (i.q.r. 31–46) minutes, respectively. ABO-incompatible grafts were used in 16.6% of patients, and hepaticojejunostomy was performed in 91.4% of patients.

**Table 1:** Baseline characteristics of recipient, donor, and graft

	Overall (*n* = 2463)	BM patients (*n* = 1988)	Non-BM patients (*n* = 475)	P*
Age (months), median (i.q.r.)	11 (6–34)	10 (6–26)	22 (7–70)	< 0.001
**Sex**				0.268
Male	1252 (50.8%)	998 (50.2%)	252 (53.0%)	
Female	1211 (49.2%)	990 (49.8%)	223 (47.0%)	
Weight (kg), median (i.q.r.)	8.2 (6.6–13.0)	8.0 (6.6–11.9)	10.0 (6.1–18.8)	< 0.001
Height (cm), median (i.q.r.)	71 (65–91)	70 (65–86)	80 (63–114)	< 0.001
PVT before transplant	38 (1.5%)	0 (0.0%)	38 (8.0%)	< 0.001
Congenital heart disease	40 (1.6%)	0 (0.0%)	40 (8.4%)	< 0.001
Hepatopulmonary syndrome	16 (0.6%)	0 (0.0%)	16 (3.4%)	< 0.001
Dialysis before transplant	61 (2.5%)	0 (0.0%)	61 (12.8%)	< 0.001
**Aetiology of liver disease**				< 0.001
Biliary atresia	1471 (59.7%)	1367 (68.8%)	104 (21.9%)	
Other cholestatic diseases	298 (12.1%)	264 (13.3%)	34 (7.2%)	
Metabolic diseases	382 (15.5%)	357 (18.0%)	25 (5.3%)	
PALF	86 (3.5%)	0 (0.0%)	86 (18.1%)	
Tumour	93 (3.8%)	0 (0.0%)	93 (19.6%)	
Other	133 (5.4%)	0 (0.0%)	133 (28.0%)	
Kasai procedure in biliary atresia	1103 of 1471 (75.0%)	1018 of 1367 (74.5%)	85 of 104 (81.7%)	< 0.001
PELD score, median (i.q.r.)	16 (7–24)	16 (8–23)	13 (0–24)	0.002
BM patients	1988 (80.7%)	1988 (100.0%)	0 (0%)	
**Donor**				
Age (years), median (i.q.r.)	32 (29–36)	32 (29–36)	34 (30–38)	< 0.001
Sex				0.396
Male	871 (43.1%)	725 (43.5%)	146 (40.9%)	
Female	1592 (56.9%)	1263 (56.5%)	329 (59.1%)	
BMI (kg/m^2^), median (i.q.r.)	23.0 (21.0–24.8)	23.0 (21.0–24.8)	23.0 (20.6–25.2)	0.795
**Graft type**				< 0.001
Left lateral segment graft	2182 (88.6%)	1808 (90.9%)	374 (78.7%)	
Left lobe graft	240 (9.7%)	157 (7.9%)	83 (17.8%)	
Right lobe graft	41 (1.6%)	23 (1.2%)	18 (3.5%)	
Graft weight (g), median (i.q.r.)	257 (220–300)	257 (220–300)	257 (215–316)	0.464
GRWR, median (i.q.r.)	2.9 (2.0–3.8)	3.0 (2.2–3.8)	2.6 (1.6–3.8)	< 0.001
Cold ischaemia time (min), median (i.q.r.)	34 (21–84)	29 (20–71)	68 (29–138)	< 0.001
Warm ischaemia time (min), median (i.q.r.)	37 (31–46)	37 (31–45)	38 (33–49)	0.013
ABO incompatibility	326 (15.5%)	286 (16.6%)	40 (10.8%)	0.007
Hepaticojejunostomy	1833 (90.6%)	1523 (91.4%)	310 (86.8%)	0.01

Values are *n* (%) unless otherwise stated. BM, benchmark; i.q.r., interquartile range; PVT, portal vein thrombosis; PALF, paediatric acute liver failure; PELD, paediatric end-stage liver disease; BMI, body mass index; GRWR, graft-to-recipient weight ratio; min, minutes. *Continuous variables were compared using the Mann–Whitney *U* test, whereas categorical variables were analysed using χ^2^ tests.

### Benchmark cut-off values and comparison with control groups

Benchmark cut-off values are summarized in *Table 2*. Cut-off values were set as follows: operation duration ≤ 8 hours, intraoperative red blood cell (RBC) transfusion ≤ 300 ml, and a length of hospital stay ≤ 40 days. One-year surgical complication thresholds included bowel perforation ≤ 5%, intra-abdominal bleeding ≤ 8%, any biliary complication ≤ 18%, anastomotic stricture ≤ 6%, bile leak ≤ 12%, hepatic artery thrombosis ≤ 5%, and portal vein thrombosis ≤ 5%. The overall benchmark for major complications was set at ≤ 50%, with 1-year graft loss and mortality both capped at ≤ 6%. For interpretability in this weight-diverse paediatric cohort, the intraoperative RBC transfusion benchmark of ≤ 300 ml corresponds to approximately ≤ 37 ml/kg at the median recipient weight of 8.0 kg. For comparison, non-benchmark cut-off values were also derived for high-risk non-benchmark patients: RBC transfusion ≤ 420 ml, length of hospital stay ≤ 50 days, bowel perforation ≤ 8%, bile leak ≤ 14%, hepatic artery thrombosis ≤ 6%, major complications ≤ 65%, 1-year graft loss ≤ 10%, and 1-year mortality ≤13% (*Table 2*). When benchmark cut-off values were applied to other settings, benchmark patients from low-volume centres exceeded thresholds for biliary complications, hepatic artery thrombosis, and portal vein thrombosis, resulting in a higher major complication rate (58%) and graft loss rate (9%). Non-benchmark patients showed significantly worse outcomes, including higher intraoperative RBC transfusions, longer lengths of hospital stay, and increased rates of bowel perforation, biliary complications, hepatic artery thrombosis, major complications, graft loss, and 1-year mortality. Among high-risk subgroups, paediatric acute liver failure (PALF) and dialysis-dependent patients had higher graft loss and mortality without a corresponding rise in surgical complications, whereas patients weighing < 5 kg had increased rates of bowel perforation (12%), intra-abdominal bleeding (10%), and portal vein thrombosis (6%). Cold ischaemia time was longer in non-benchmark than benchmark patients (median 68 *versus* 29 minutes; *Table 1*), reflecting the greater proportion of larger left-lobe and right-lobe grafts requiring more extensive back-table preparation, together with the higher prevalence of portal vein thrombosis and previous abdominal surgery, which prolong the recipient hepatectomy and vascular reconstruction. All outcome comparisons, including those for the PALF and dialysis-dependent subgroups, were based exclusively on LDLT recipients (*Table 2*).

**Table 2:** Outcome comparisons between BM and higher-risk cohorts

Perioperative course	BM cut-off value	BM cohorts from low-volume centres (*n* = 124)	Paediatric acute liver failure (*n* = 102)	Weight < 5 kg (*n* = 89)	Dialysis dependant (*n* = 65)	Non-BM cut-off value
Operation duration (h), median (i.q.r.)	≤ 8	7.9 (6.2–9.6)	6.5 (5.5–7.6)	6.7 (5.4–7.8)	7.0 (6.0–7.7)	≤ 8
Intraopertaive RBC transfusion (ml), median (i.q.r.)	≤ 300	**350 (200–600)**	**360 (200–500)**	300 (200–400)	**375 (200–560)**	**≤** **420**
LOS (days) , median (i.q.r.)	≤ 40	30 (22–44)	27.5 (19–50)	30 (19–48)	**46** (**22–72)**	**≤** **50**
**Negative events at 1 year**						
Bowel perforation	≤ 5%	4 (3.2%)	4 (4.0%)	**10 (11.9%)**	**5 (7.7%)**	**≤** **8%**
Intra-abdominal bleeding	≤ 8%	8 (6.5%)	8 (7.9%)	**8 (9.5%)**	4 (6.2%)	≤ 8%
Any biliary complication	≤ 18%	**24 (19.4%)**	14 (13.7%)	2 (2.2%)	4 (6.2%)	**≤** **20%**
Anastomotic strictures	≤ 6%	7 (5.6%)	3 (2.9%)	0 (0%)	1 (1.5%)	≤ 6%
Bile leak	≤ 12%	**16 (12.9%)**	10 (9.8%)	2 (2.2%)	3 (4.6%)	**≤** **14%**
Hepatic artery thrombosis	≤ 5%	**7 (5.6%)**	3 (2.9%)	3 (3.4%)	1 (1.5%)	**≤** **6%**
Portal vein thrombosis	≤ 5%	**10 (8.1%)**	4 (3.9%)	**5 (5.6%)**	2 (3.1%)	≤ 5%
**Morbidity and mortality at 1 year**						
Readmission	≤ 50%	27 (21.8%)	19 (18.8%)	16 (19.0%)	11 (16.9%)	≤ 50%
Grade ≥ 3a complication	≤ 50%	**72 (58.1%)**	51 (49.5%)	42 (50.0%)	**37 (56.9%)**	**≤** **65%**
Graft loss	≤ 6%	**11 (8.9%)**	**11 (10.9%)**	**7 (8.7%)**	**14 (21.5%)**	**≤** **10%**
Mortality/grade 5 complication	≤ 6%	7 (5.6%)	**13 (12.9%)**	**11 (13.1%)**	**12 (18.5%)**	**≤** **13%**

Values are *n* (%) unless otherwise stated. BM cut-off values are defined by the 75th percentile of centre medians. All complications were graded using the Clavien–Dindo classification system. Bold text indicates values above the benchmark cut-offs. BM, benchmark; h, hours; i.q.r., interquartile range; LOS, length of hospital stay.

### Impact of centre volume on outcomes

**Figure 2:**
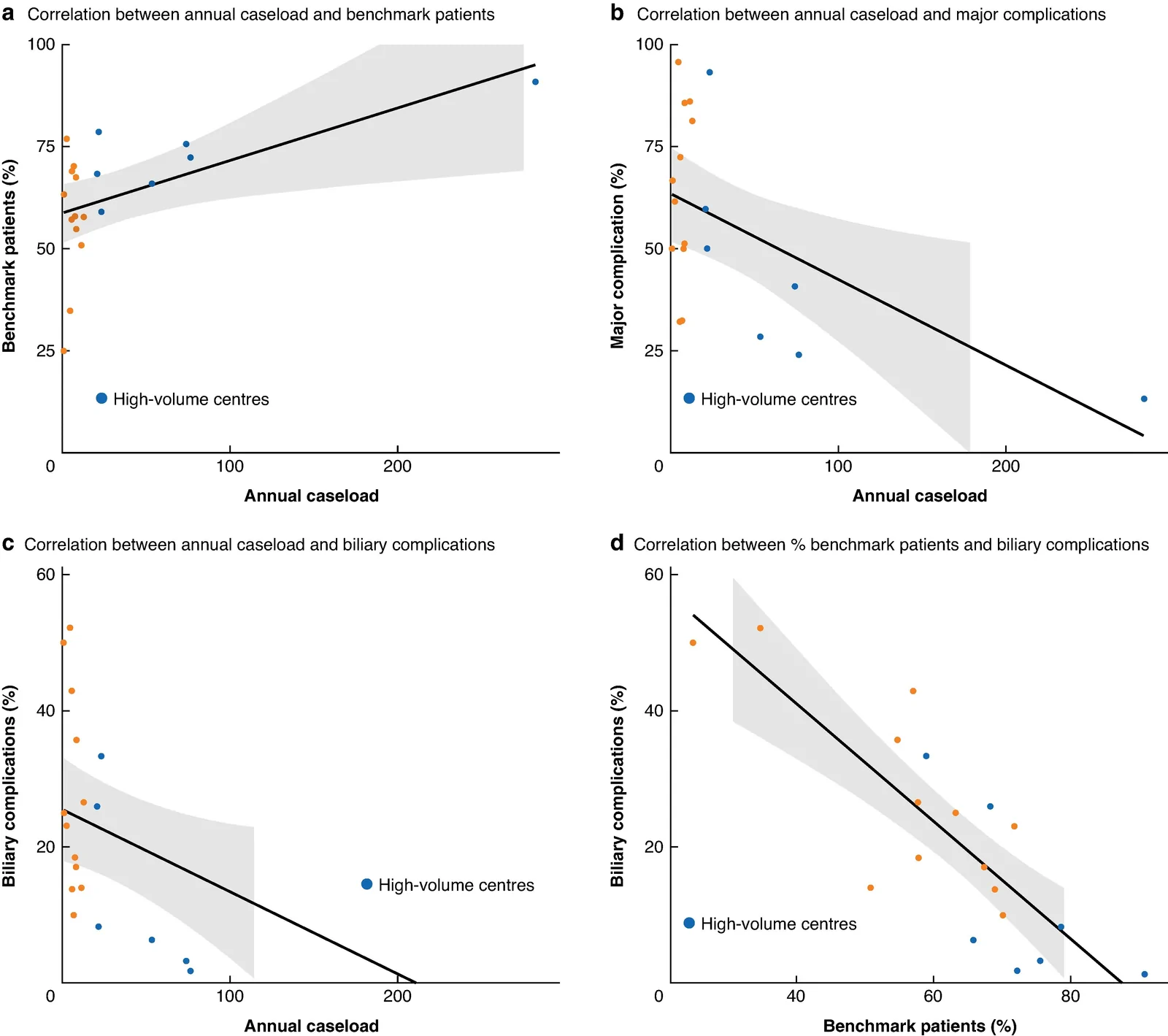
Linear correlations between centre characteristics and clinical outcomes Scatter plots demonstrating correlations between the annual caseload and **a** the proportion of benchmark patients (*R*^2^ = 0.55; *P* = 0.01), **b** the incidence of major complications within 1 year (*R*^2^ = –0.55; *P* = 0.01), and **c** the incidence of biliary complications (*R*^2^ = –0.50; *P* = 0.03), as well as **d** the correlation between the proportion of benchmark patients and the incidence of biliary complications (*R*^2^ = –0.83; *P* < 0.001). Linear correlations are presented as *R*^2^ values, with *P* < 0.05 considered statistically significant. Shaded areas indicate 95% confidence intervals.

Although modest, a higher annual caseload was associated with a greater proportion of benchmark patients, as well as lower rates of major complications, particularly biliary complications (*Fig. 2a–c*). Notably, the proportion of benchmark patients showed a strong inverse correlation with the rate of biliary complications (*R*^2^ = −0.83, *P* < 0.001; *Fig. 2d*). Annual caseload was also inversely correlated with failure-to-rescue rates (*Fig. S2*).

### Comparison of grade III complication classification between the Clavien–Dindo and Clavien–Madadi systems

**Figure 3:**
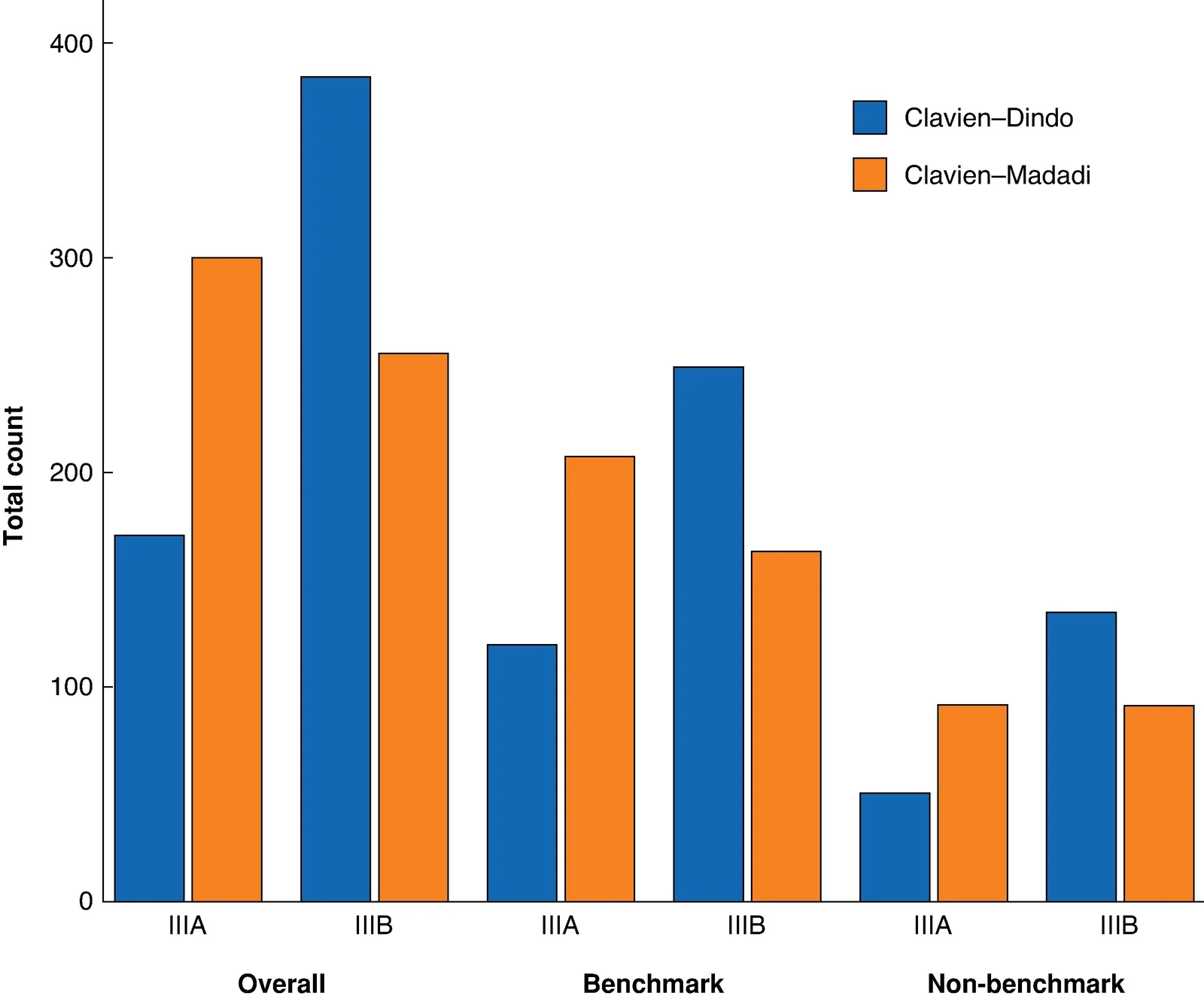
Comparison of complication grading between Clavien–Dindo and Clavien–Madadi classifications Bar plots show the absolute counts of major complications in paediatric living donor liver transplantation using the Clavien–Dindo and Clavien–Madadi classification systems.

When classifying post-transplant complications using the Clavien–Dindo system, most grade III events were categorized as grade IIIB (more than twice as many as grade IIIA). In contrast, under the Clavien–Madadi system, many complications previously labelled as grade IIIB by the Clavien–Dindo system were reclassified as grade IIIA. As a result, grade IIIB complications were less common in the Clavien–Madadi classification (*Fig. 3*). This pattern was observed not only in the overall population but also consistently across both benchmark and non-benchmark patients.

## Discussion

This multicentre study defines key benchmarks for the paediatric LDLT. Although benchmark cut-off values represent the best achievable outcomes in ideal patients, comparative data remain limited because most registries do not report graft type-specific complication rates. One exception is the SPLIT registry, which reports comparable rates of hepatic artery thrombosis (8%) and portal vein stenosis (4.4%)^21^. However, the overall biliary complication rate (24%) in the SPLIT registry exceeds the benchmark cut-off values determined in the present study (≤ 12% leak, ≤ 6% stricture). This discrepancy likely reflects cohort differences: the present benchmark cohort comprised low-risk LDLT patients from high-volume centres, whereas the SPLIT registry includes both deceased and living donor grafts across varied centre volumes, potentially encompassing higher-risk patients and less experienced programmes. Furthermore, SPLIT includes all biliary complications, whereas the present study specifically analysed bile leak and stricture as distinct endpoints. Similarly, 1-year LDLT graft loss rates from the European Registry (14%)^16^ and national data from Japan^15,22^ and Korea (∼10%)^23^ exceed the present study’s benchmark of 6%, likely reflecting the inclusion of higher-risk patients. Collectively, these findings support the attainability and clinical relevance of benchmark outcomes in select patients. Notably, low-volume centres, despite evaluating benchmark patients, failed to meet critical cut-off values for biliary and vascular complications, as well as graft loss, suggesting a need for enhanced surgical expertise through dedicated workshops and exchanges with high-volume centres. However, mortality rates at low-volume centres remained within benchmark limits, implying that adequate organ support compensated for graft losses. Analysis of non-benchmark patients showed that PALF or dialysis-dependent patients had high mortality independent of surgical complications. Conversely, low-weight patients were more susceptible to vascular complications, likely due to smaller anatomical structures. Consequently, defining standard outcomes for non-benchmark patients necessitates consideration of their specific underlying conditions.

Centre volume also correlated with superior outcomes. Higher annual caseload was inversely correlated with failure to rescue, suggesting high-volume centres manage major complications more successfully when they occur. This aligns with studies of SRTR^24–26^ and SPLIT^27^ registries, the latter identifying failure to rescue as a key determinant of patient and graft survival. However, low-volume centres managed a higher prevalence of complex, non-benchmark patients (*Table S1*). Although larger programmes may be expected to handle more high-risk patients, this inverse distribution suggests that current case allocation may be driven by regional referral networks rather than expertise alone, highlighting a need for optimized referral pathways. This pattern is consistent with structural rather than expertise-based allocation: most high-volume centres in the present study cohort operate within centralized or regionalized national systems, whereas the lower-volume centres are predominantly from countries with dispersed transplant networks, where standard-risk patients are distributed across many programmes and high-risk patients are referred to tertiary centres that remain below the volume threshold applied here. Accordingly, safe expansion of LDLT is most realistically achieved by beginning with low-risk, benchmark-eligible recipients, regionalizing higher-risk patients to experienced centres, and supporting emerging programmes through proctoring and prospective benchmarking, rather than by requiring a fixed annual volume at every centre.

Importantly, benchmarking is not intended to discourage transplantation in higher-risk paediatric populations or penalize centres caring for complex patients. Benchmark cut-off values are derived exclusively from ideal, low-risk patients to define best achievable outcomes, serving as a quality reference for surgical technique rather than an assessment of overall programme performance. Although separate benchmarks could be developed for specific high-risk subgroups, this approach would be impractical for broad application. Instead, benchmarking should complement existing systems evaluating overall institutional performance and case mix.

This study used the novel Clavien–Madadi classification^12^, developed for paediatric surgical procedures. The Clavien–Dindo system tends to overclassify grade IIIB complications in children because it relies on anaesthetic management, even though general anaesthesia is often required for minor procedures in paediatric patients. In contrast, the Clavien–Madadi system focuses on the invasiveness of the intervention, revealing that Clavien–Dindo grade IIIB events were not truly major procedures (for example, laparotomies or thoracotomies). Validated only in adult transplantation, the CCI^®^ warrants validation for the paediatric population.

This study has several limitations. First, its retrospective design introduces inherent biases. Second, no consensus defines ‘high-volume’ centres for paediatric LDLT. The threshold used in the present study (≥ 20 cases per year) was chosen based on the natural cohort distribution. This higher cut-off was selected to ensure the benchmarks reflect highly experienced programmes. As a consequence of this dichotomy, intermediate-volume centres were not represented, and their outcomes, which may differ from both high- and low-volume programmes, warrant dedicated study. Furthermore, paediatric LDLT volumes decreased due to COVID-19 in 2020, potentially preventing some centres from reaching this threshold. Third, high-volume centres contributed most patients, which may bias findings towards larger centres. In particular, the single largest centre contributed 45% of the cohort, so the descriptive characteristics and pooled estimates are weighted towards its practice; the benchmark thresholds themselves are less affected, because they were derived from centre-level medians that weight each centre equally. Furthermore, biliary complication rates varied widely among lower-volume centres, and the association with higher volume was influenced by a single high-volume outlier (*Fig. 2c*), limiting generalizability. In addition, this study represents the first application and comparative evaluation of the Clavien–Madadi classification in paediatric LDLT rather than a formal validation against an external reference standard, which remains to be performed. The benchmark methodology is descriptive by design and was not intended to derive a multivariable risk-prediction model; formal identification of additional independent outcome determinants would require dedicated modelling. In addition, because the cohort comprised LDLT recipients exclusively, practice could not be contextualized against the proportion of deceased donor paediatric transplantation, which is better addressed using registry data. Finally, although the international scope provided a large and diverse study population, it introduced variability in diagnostic criteria and surgical techniques. Future research should focus on validating these benchmark cut-off values in large transplant registries and assessing their impact on clinical practice.

## Supplementary Material

zrag154_Supplementary_Data

## Data Availability

The data that support the findings of this study are not publicly available due to privacy and ethical restrictions but are available from the corresponding author upon reasonable request and with appropriate institutional approval.
