# Supplementary material for: Benchmarking paediatric living donor liver transplantation for establishing standards to expand access and save lives: retrospective global cohort study
Source: BJS Open. 2026 Sep 25;10(5):zrag154. doi: 10.1093/bjsopen/zrag154 (PMC13614256; doi:10.1093/bjsopen/zrag154)
Supplement: Supplementary file 1 [file zrag154_supplementary_data.docx]

**Benchmarking Pediatric Living Donor Liver Transplantation: Establishing Standards to Expand Access and Safe Lives: retrospective global cohort study**

Zhihao Li¹, Omid Madadi-Sanjani², Yuan Liu³, Dimitri A. Raptis⁴, Vasanthakumar Gunasekaran⁵, Ashwin Rammohan⁵, Seisuke Sakamoto⁶, Hajime Uchida⁶, Piotr Kaliciński⁷, Marek Stefanowicz⁷, Nicolas Goldaracena⁸, Cornelius J. van Beekum⁹, Felix J. Krendl¹⁰, Itsuko Chih-Yi Chen¹¹, Bryanna N. Domenick¹², Alfred Kow¹³, Kaihang Wang¹⁴, Yuji Soejima¹⁵, Katryn N. Furuya¹⁶, George V. Mazariegos¹⁷, Vidyadhar Padmakar Mali¹³, Stephen Dunn¹⁸, Kim Olthoff¹²˒¹⁹, Elizabeth B. Rand¹², Ian Leipnitz²⁰, Chao-Long Chen¹¹, Deniz Balci²¹, Elvan Onur Kirimker²², Silvio Nadalin²³, Stefan Schneeberger¹⁰, Debra L. Sudan²⁴, Moritz Schmelzle⁹, Uta Herden², Lutz Fischer², Blayne A. Sayed⁸˒²⁵, Vicky L. Ng²⁵, Grzegorz Kowalewski⁷, Mureo Kasahara⁶, Mohamed Rela⁵, Dieter C. Broering⁴, Qiang Xia³, Pierre-Alain Clavien¹˒²⁶

1. Department of Surgery and Transplantation, University of Zurich, Zurich, Switzerland
2. Department of Visceral Transplantation, University Medical Center Hamburg-Eppendorf, Hamburg, Germany
3. Department of Liver Surgery, Renji Hospital Affiliated to Shanghai Jiao Tong University School of Medicine, Shanghai, China
4. King Faisal Specialist Hospital & Research Centre, Riyadh, Saudi Arabia
5. Dr Rela Institute and Medical Center, Chennai, India
6. Organ Transplantation Center, National Center for Child Health and Development (NCCHD), Tokyo, Japan
7. Department of Pediatric Surgery and Organ Transplantation, The Children's Memorial Health Institute, Warsaw, Poland
8. Department of Surgery, Division of Abdominal Transplant Surgery, University Health Network, Toronto, Ontario, Canada
9. Department of General, Visceral and Transplant Surgery, Hannover Medical School, Hannover, Germany
10. Department of Visceral, Transplant and Thoracic Surgery, Center for Operative Medicine, Medical University of Innsbruck, Innsbruck, Austria
11. Department of Surgery, Kaohsiung Chang Gung Memorial Hospital, Kaohsiung, Taiwan
12. Children's Hospital of Philadelphia, Philadelphia, Pennsylvania, USA
13. Department of Paediatric Surgery, National University Hospital, Singapore
14. University of Zurich, Zurich, Switzerland
15. Division of Gastroenterological, Hepato-Biliary-Pancreatic, Transplantation and Pediatric Surgery, Department of Surgery, Shinshu University School of Medicine, Matsumoto, Japan
16. Department of Pediatrics, University of Wisconsin-Madison School of Medicine and Public Health, Madison, Wisconsin, USA
17. Hillman Center for Pediatric Transplantation, UPMC Children's Hospital of Pittsburgh, Pittsburgh, PA, USA
18. Department of Solid Organ Transplantation, Alfred I. duPont Hospital for Children, Wilmington, DE, USA
19. Department of Surgery, Penn Transplant Institute, Perelman School of Medicine, University of Pennsylvania, Philadelphia, Pennsylvania, USA
20. Liver Transplant Unit, University of Auckland, Auckland, New Zealand
21. Bahçeşehir University Faculty of Medicine, Department of General Surgery, İstanbul, Turkey
22. Ankara University Faculty of Medicine, Department of General Surgery, Ankara, Turkey
23. Eberhard Karls University of Tübingen, Department of General Visceral and Transplant Surgery, Tübingen, Germany
24. Division of Abdominal Transplantation, Department of Surgery, Duke University Medical Center, Durham, North Carolina, USA
25. The Hospital for Sick Children, Toronto, ON, Canada
26. Wyss Zurich Translational Center, ETH Zurich and University of Zurich, Zurich, Switzerland

**Corresponding author.**

Zhihao Li, MD

University of Zurich, Rämistrasse 71, CH-8006 Zurich,

Email: [zhi-hao.li@outlook.com](mailto:zhi-hao.li@outlook.com)

**Supplementary Materials - Index**

| **Supplementary Figures and Tables** |  |
| --- | --- |
| SUPPLEMENTARY TABLE 1 | *page 3* |
| SUPPLEMENTARY FIGURE 1 | *page 4* |
| SUPPLEMENTARY FIGURE 2 | *page 5* |
|  |  |

**Supplementary Figures and Tables**

| **SUPPLEMENTARY TABLE S1:** Baseline Characteristics of Recipient, Donor and Graft in High- and Low-Volume Centers. | | | |
| --- | --- | --- | --- |
| **Characteristics** | **High-volume Center (n=2463)** | **Low-volume Center (n=202)** | **P-value** |
| Age, months, median [IQR] | 11 [6-34] | 12 [7-51] | 0.041 |
| Sex, male, n (%) | 1252 (50.8) | 95 (47.0) | 0.343 |
| Weight, kg, median [IQR] | 8.2 [6.6-13.0] | 8.8 [6.6-15.0] | 0.165 |
| Height, cm, median [IQR] | 71 [65-91] | 75 [65-102] | 0.068 |
| PVT pretransplant, n (%) | 38 (1.5) | 11 (5.4) | <0.001 |
| Congenital heart disease, n (%) | 40 (1.6) | 10 (5.0) | 0.002 |
| Hepatopulmonary syndrome, n (%) | 16 (0.6) | 3 (1.5) | 0.355 |
| Dialysis pretransplant, n (%) | 61 (2.5) | 4 (2.0) | 0.842 |
| Etiology of liver disease, n (%) |  |  | <0.001 |
| Biliary atresia | 1471 (59.7) | 117 (57.9) |  |
| Other cholestatic diseases | 298 (12.1) | 17 (8.4) |  |
| Metabolic diseases | 382 (15.5) | 20 (9.9) |  |
| Pediatric acute liver failure | 86 (3.5) | 16 (7.9) |  |
| Tumor | 93 (3.8) | 9 (4.5) |  |
| Others | 133 (5.4) | 23 (11.4) |  |
| Kasai procedure in biliary atresia, n (%) | 1103/1471 (75.0) | 103/117 (88.0) | 0.217 |
| PELD score, median [IQR] | 16 [7-24] | 17 [8-25] | 0.346 |
| Benchmark patient, n (%) | 1988 (80.7) | 124 (61.7) | <0.001 |
| Age donor, years, median [IQR] | 32 [29-36] | 33 [28-38] | 0.307 |
| Sex donor, male, n (%) | 871 (43.1) | 86 (42.6) | 0.955 |
| BMI donor, kg/m2, median [IQR] | 23.0 [21.0-24.8] | 24.3 [22.5-27.2] | <0.001 |
| Graft type, n (%) |  |  | 0.061 |
| Left lateral segment graft | 2182 (88.6) | 169 (83.7) |  |
| Left lobe graft | 240 (9.7) | 27 (13.4) |  |
| Right lobe graft | 41 (1.6) | 6 (3.0) |  |
| Graft weight, g, median [IQR] | 257 [220-300] | 267.00 [229.75, 320.00] | 0.016 |
| GRWR, median [IQR] | 2.9 [2.0-3.8] | 3.0 [1.9-4.0] | 0.552 |
| Cold ischemia time, min, median [IQR] | 34 [21-84] | 55 [34-132] | <0.001 |
| Warm ischemia time, min, median [IQR] | 36 [31-45] | 40 [32-49] | 0.013 |
| ABO-incompatibility, n (%) | 326 (15.5) | 14 (7.1) | 0.002 |
| Hepatico-jejunostomy, n (%) | 1833 (90.6) | 169 (84.1) | 0.005 |
| Abbreviations: BMI, Body mass index; GRWR, Graft-to-Recipient weight ratio; PELD, Pediatric End-Stage Liver Disease; PVT, Portal vein thrombosis. | | | |

**SUPPLEMENTARY FIGURE S1:** **Distribution of Benchmark Cases Across Contributing Centers.** The proportion of benchmark cases is expressed as a percentage of total cases. Black bars represent the absolute number of benchmark cases, and grey bars represent the absolute number of total cases at each center. The proportion of benchmark cases is expressed as a percentage of total cases performed at each center.

**SUPPLEMENTARY FIGURE S2:** Linear Correlations Between Annual Caseload and Failure to Rescue Rates.
